# Prehabilitation Before Cardiac Surgery and Structural Heart Interventions: An Umbrella Review of Pooled Evidence

**DOI:** 10.3390/jcm15103821

**Published:** 2026-05-15

**Authors:** Elen H. Hughes, Robyn Lotto, Ellen A. Dawson, Mohamed Saber, Ethan Richards, Adrian Morris, David Mayhew, Fahmi Faraz, Reza Ashrafi, Julia D. Jones

**Affiliations:** 1 Liverpool Heart and Chest Hospital NHS Foundation Trust, Liverpool L14 3PE, UK; 2 Research Institute for Sport and Exercise Science, Faculty of Health, Innovation, Technology & Science, Liverpool John Moores University, Liverpool L3 3AF, UK; 3 Liverpool Centre for Cardiovascular Science, Liverpool L7 8TX, UK; 4 School of Nursing, Public and Allied Health, Faculty of Health, Innovation, Technology & Science, Liverpool John Moores University, Liverpool L3 3AF, UK; 5 School of Medicine, Faculty of Medical Sciences, Newcastle University, Newcastle NE2 4HH, UK

**Keywords:** prehabilitation, cardiac interventions, cardiac surgery, exercise training, inspiratory muscle training, umbrella review

## Abstract

**Background**: Prehabilitation aims to optimise patients before cardiac procedures through interventions including exercise training, respiratory conditioning, nutritional support, psychological preparation and multimodal lifestyle programmes. Evidence from systematic reviews and meta-analyses is increasing but remains heterogeneous due to variation in intervention design, patient populations and overlap of primary studies. **Methods**: We conducted an umbrella review of 17 systematic reviews and meta-analyses evaluating prehabilitation prior to cardiac surgery and structural heart interventions in accordance with the Preferred Reporting Items for Systematic Reviews and Meta-Analyses (PRISMA) guidelines. Methodological quality of included reviews was assessed using A Measurement Tool to Assess Systematic Reviews 2 (AMSTAR 2). Outcomes of interest were postoperative pneumonia, hospital length of stay (LOS), and mortality. **Results**: Across pooled analyses, the most consistent finding was a reduction in postoperative pneumonia, particularly in studies incorporating inspiratory muscle training (IMT), with relative risk reductions of approximately 55–62%, corresponding to a modest absolute risk reduction. Reductions in hospital LOS were also reported, although effect sizes were smaller and more variable. In contrast, no consistent reduction in short-term mortality was demonstrated, likely reflecting low event rates. The evidence base was limited by substantial overlap between reviews and predominantly low or critically low methodological quality. **Conclusions**: Prehabilitation, particularly when incorporating IMT, is consistently associated with a reduction in postoperative pneumonia and may contribute to modest reductions in hospital LOS. However, the evidence base is constrained by heterogeneity, study overlap and low methodological quality. Further high-quality, adequately powered randomised trials are required to define the role of prehabilitation in contemporary cardiac surgical and structural intervention practice.

## 1. Introduction

Advances in cardiac surgery and interventional cardiology have expanded therapeutic options across a broad spectrum of cardiovascular disease. Contemporary patient populations undergoing these procedures are increasingly characterised by a high burden of comorbidity, including frailty, sarcopenia, obesity and cardiometabolic disease, all of which influence perioperative risk and postoperative outcomes [1,2].

Prehabilitation encompasses a broad spectrum of approaches, ranging from single-component interventions, such as inspiratory muscle training (IMT) or structured exercise, to multimodal strategies. These interventions are designed to enhance physiological reserve and perioperative resilience, supporting postoperative recovery [3].

Exercise-based prehabilitation typically involves structured aerobic and/or resistance training to improve functional capacity and cardiopulmonary reserve. Programmes may be supervised or home-based and vary in intensity and mode of delivery [4,5]. IMT is a targeted respiratory intervention involving threshold-loaded breathing exercises that provide resistance during inhalation. It aims to strengthen the respiratory muscles, improve ventilatory efficiency, and enhance postoperative respiratory reserve [6].

A growing number of systematic reviews and meta-analyses have evaluated prehabilitation in patients undergoing cardiac surgery and structural heart interventions, but the evidence remains heterogeneous, reflecting variation in patient populations, intervention design, definitions, and outcome reporting. Despite increasing interest, this heterogeneity constrains meaningful synthesis and interpretation [7,8]. Recent work has highlighted the absence of a standardised definition of prehabilitation and variability in how interventions are delivered across studies, contributing to low certainty of evidence [9]. Furthermore, real-world data suggest that prehabilitation programmes are increasingly implemented despite this uncertainty, with considerable variation in provision and practice across centres [10]. In cardiac surgery specifically, existing trials have largely focused on single-component interventions and small study populations, and there remains a lack of adequately powered, definitive studies to guide clinical practice [11].

This umbrella review aims to synthesise existing evidence on the effects of prehabilitation across three key outcomes: postoperative pneumonia, hospital length of stay (LOS) and mortality in patients undergoing cardiac surgical and structural interventions.

These outcomes were selected based on clinical relevance, frequency of reporting in the literature and objectivity. Postoperative pneumonia represents a clinically important complication associated with increased morbidity and prolonged recovery [12]. Hospital LOS is an easily quantifiable and widely reported measure of postoperative recovery and healthcare utilisation, while mortality provides a binary, objective outcome. Together, these outcomes capture complementary aspects of perioperative risk, recovery, and overall clinical effectiveness.

## 2. Materials and Methods

### 2.1. Study Design

This umbrella review was conducted in accordance with the Preferred Reporting Items for Systematic Reviews and Meta-Analyses 2020 (PRISMA 2020) guidelines (Appendix A), using a structured and predefined methodological approach [13,14]. The study addresses a priority identified by the James Lind Alliance for anaesthesia and perioperative care, specifically “How can preoperative exercise or fitness training, including physiotherapy, improve outcomes after surgery?” [15]. The protocol was subsequently registered on the Open Science Framework (OSF) (https://osf.io/tzkeh). During the preparation of this manuscript, the authors used ChatGPT 5.0 for the purposes of improving the clarity and language of the manuscript. The authors have reviewed and edited the output and take full responsibility for the content of this publication.

### 2.2. Eligibility Criteria

Eligible studies were systematic reviews and meta-analyses evaluating prehabilitation interventions in adult patients aged ≥18 years undergoing elective cardiac surgery or structural heart interventions, excluding cardiac transplantation, where prehabilitation was delivered prior to the planned procedure. Prehabilitation was defined as an intervention including at least one active physiological component, such as exercise training or IMT. Included reviews were required to report pooled analyses for postoperative pneumonia, hospital LOS, or mortality. Reviews were excluded if pooled analyses included postoperative interventions and the effects of preoperative components were not clearly reported.

### 2.3. Search Strategy

A systematic literature search was conducted across three databases, PubMed, Embase and the Cochrane Central Register of Controlled Trials, from database inception to March 2026. The search strategy combined terms related to prehabilitation, preoperative exercise, respiratory muscle training and physiotherapy, combined with terms relating to cardiac surgery and structural heart interventions. The full search string for each database is provided in Appendix B.

### 2.4. Study Selection and Data Extraction

All identified records were imported into EndNote 2025 (Clarivate, Philadelphia, PA, USA), and duplicates were removed. Titles and abstracts were screened by a single reviewer (E.H.), with a 25% sample independently being evaluated by a second reviewer (J.J.) to ensure consistency of study selection. While this approach reflects a pragmatic balance between methodological rigour and feasibility, it may introduce a degree of selection bias. Formal inter-rater agreement (e.g., kappa statistic) was not calculated. Articles selected for inclusion underwent full-text assessment by two reviewers (R.L. and E.H.), with disagreements being resolved by a third reviewer (J.J.).

Data extraction was performed using a standardised approach and independently cross-checked by a second reviewer (M.S.). Extracted data included the number of studies and participants, type of cardiac surgery or intervention, prehabilitation modality, outcomes assessed and reported effect measures.

### 2.5. Quality Assessment

The methodological quality of the included systematic reviews was assessed using the AMSTAR 2 (A Measurement Tool to Assess Systematic Reviews 2) checklist [16]. Two reviewers (R.L. and E.R.) independently evaluated each review across all 16 domains, rating items as ‘Yes,’ ‘Partial,’ or ‘No’ in accordance with AMSTAR 2 guidance.

Discrepancies were resolved through discussion and consensus. Overall confidence ratings (high, moderate, low, or critically low) were assigned based on the presence of critical and non-critical weaknesses, following AMSTAR 2 recommendations. An informal narrative sensitivity assessment was undertaken by comparing effect estimates from reviews rated high- or moderate-quality using AMSTAR 2.

### 2.6. Certainty of Evidence Assessment

The certainty of evidence for each outcome was assessed using the GRADE approach (Grading of Recommendations Assessment, Development and Evaluation) [17]. Given substantial overlap in primary studies across the included systematic reviews, GRADE assessments were not performed for every pooled analysis, to avoid repeated evaluation of the same underlying evidence. Instead, a single representative meta-analysis was selected for each outcome using a hierarchical approach based on: (1) methodological quality according to AMSTAR 2; (2) relevance to the review question, with preference for cardiac-only populations where available; (3) clarity and completeness of outcome reporting; and (4) completeness and clinical relevance of the pooled dataset. More recent meta-analyses were prioritised, where methodologically appropriate, to enhance relevance to contemporary clinical practice. For example, Cursino de Moura et al. (2024) [18] was selected for pneumonia due to its cardiac-specific population and inclusion of more recent trials compared with earlier high-quality reviews.

This approach differs from standard GRADE application across all available meta-analyses and may introduce selection bias. However, it was adopted as a pragmatic strategy to manage substantial overlap among reviews, recognising that the included meta-analyses draw on largely overlapping but not identical sets of primary studies.

GRADE domains were applied to the body of primary-study evidence contributing to the selected meta-analysis to generate an outcome-level estimate of certainty. This approach was intended to minimise duplication while maintaining a structured and transparent assessment of available evidence.

### 2.7. Assessment of Overlap

Overlap between included systematic reviews was assessed using the corrected covered area (CCA), a measure of primary-study duplication across reviews [19].

### 2.8. Data Synthesis

Given the substantial overlap between reviews and heterogeneity in study populations, intervention design and outcome reporting, a quantitative meta-analysis was not performed. Although secondary qualitative synthesis using least-overlapping datasets could have been considered, this was not undertaken due to the complexity of overlap and heterogeneity across included reviews.

Instead, a narrative synthesis was undertaken, with results presented descriptively across included reviews. Reported effect measures (including odds ratios, relative risks, and mean differences) were extracted and summarised without conversion to a common metric, reflecting differences in reporting across systematic reviews and meta-analyses.

## 3. Results

### 3.1. Study Selection

**Figure 1:**
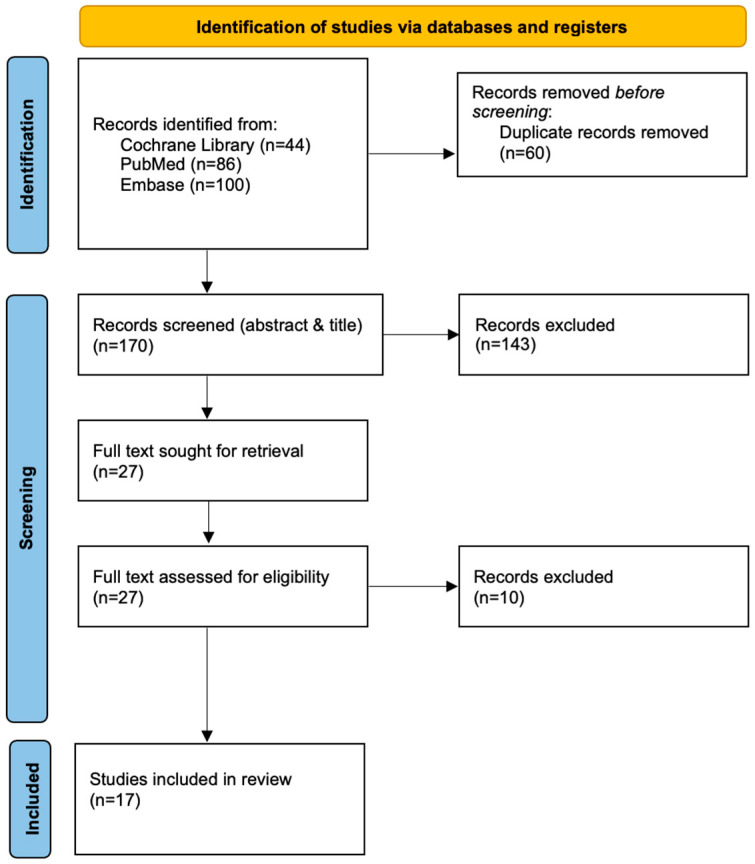
PRISMA diagram for included reviews.

A total of 230 records were identified through database searching (Cochrane: 44; PubMed: 86; Embase: 100). After removal of duplicates, 170 unique records remained for screening. Following title and abstract screening, 143 records were excluded. Twenty-seven full-text articles were sought for retrieval and assessed for eligibility, of which 10 were excluded. Seventeen were included in the final analysis (Figure 1).

### 3.2. Characteristics of Included Reviews

The characteristics of included reviews are summarised in Table 1. Overall, the evidence base comprised 17 systematic reviews, most of which also included meta-analyses. The reviews were published between 2012 and 2026.

Most reviews evaluated prehabilitation in patients undergoing elective cardiac surgery, particularly coronary artery bypass grafting (CABG) and valve surgery [4,5,7,18,20,21,22,23,24,25,26]. A smaller number also included patients undergoing elective transcatheter structural intervention [27,28,29,30]. Two reviews included mixed surgical populations rather than exclusively cardiac cohorts, although cardiac data were reported separately [31,32].

Interventions varied substantially across reviews. The most commonly evaluated intervention was IMT, either as a standalone strategy [18,20,23,24,28,31,32] or as part of a broader programme [4,5,7,21,22,25,26,27,29,30]. Other interventions included exercise-based physical therapy (PT), education, psychological support, and, in the most recent broader reviews, multimodal prehabilitation incorporating several components [4,5,7,21,22,25,26,27,29,30].

There was substantial overlap in the primary studies included across the reviews, particularly among older IMT-focused trials in CABG and valve surgery populations [21,23,31]. A total of 214 primary studies were identified across 17 reviews, of which 54 were unique. The calculated CCA was 19%, indicating a very high degree of overlap. A small number of influential primary trials contributed to multiple meta-analyses and likely represent the core evidence underpinning pooled estimates. For example, early IMT trials such as Hulzebos et al. (2006) [33] were repeatedly included across reviews. Full details of the overlap can be found in Appendix E.

**Table 1:** Characteristics of included reviews.

Study	Population	Intervention(s)	Number of Studies [n Participants]	Design	Meta-Analysis Effect Model	Heterogeneity	Outcomes Extracted	Other Reported Outcomes
**Cursino de Moura et al. (2024) [18],** **Brazil**	Elective surgery (CABG/valve)	IMT	8 [696]	Meta-analysis	Random	I^2^, X^2^	Pneumonia, total LOS, mortality	PFTs; MVT; ET/FC; PPCs
**Elbadrawy et al. (2025) [27],** **New Zealand**	Elective surgery (CABG/valve) Elective TAVI	PT, IMT, education	17 [3299]	Meta-analysis	Random	I^2^	Total LOS	ICU LOS; MVT; ET/FC; PFTs; QoL; PPCs
**Gomes Neto et al. (2017) [20],** **Brazil**	Elective surgery (CABG/valve)	IMT	8 [574] *	Meta-analysis	Random	I^2^	Total LOS	Inspiratory muscle strength; PFTs; PPCs
**Hulzebos et al. (2012) [21],** **Netherlands**	Elective surgery (CABG/valve)	PT, IMT	8 [NR]	Cochrane review	Random	I^2^, X^2^	Pneumonia, total LOS, mortality	PPCs; QoL; ET/FC; Economic costs
**Hurtado-Borrego et al. (2025) [22],** **Spain**	Elective surgery (CABG/valve)	PT, IMT	9 [873]	Meta-analysis/regression	Random	I^2^	Total LOS, mortality	ET/FC; frailty; sarcopenia; POCs; MVT; ICU LOS; QoL
**Katsura et al.** **(2015) [31],** **Japan**	Mixed surgical cohort	IMT	12 [448 estimated cardiac participants]	Cochrane review	Random	I^2^, X^2^	Pneumonia	PPCs; MVT; QoL; adverse events; PFTs; drop-out; economic costs; total LOS; mortality
**Kendall et al.** **(2018) [32],** **Portugal**	Mixed surgical cohort	IMT	NR	Meta-analysis	Random	I^2^	Total LOS	PPCs
**Marmelo et al.** **(2018) [4],** **Portugal**	Elective surgery (CABG/valve)	PT, IMT	NR	Meta-analysis	Random	I^2^	Total LOS	POCs; PFTs; ET/FC; QoL; anxiety and depression
**Rodrigues et al. (2021) [24],** **Portugal**	Elective surgery (CABG/valve)	IMT	11 [1240]	Meta-analysis	Random	I^2^	Total LOS	PPCs; PFTs
**Shahood et al.** **(2022) [26],** **Hungary**	Elective surgery (CABG/valve)	PT, IMT, education	10 [1458]	Meta-analysis	Random	I^2^	Total LOS	PPCs; PFTs; surgery time; ICU LOS; MVT
**Snowdon et al.** **(2014) [25],** **Australia**	Elective surgery (CABG/valve)	PT, IMT, education	17 [2689]	Meta-analysis	Fixed + random	I^2^	Total LOS	PPCs; MVT; ICU LOS; economic costs
**Steinmetz et al. (2023) [7],** **Germany**	Elective surgery (CABG/valve)	PT, IMT, education	6 [665]	Meta-analysis	Random	I^2^	Pneumonia, total LOS, mortality	ET/FC; ICU LOS; POCs; economic costs
**Steinmetz et al. (2026) [29],** **Germany**	Elective surgery (CABG/valve) Elective TAVI	PT, IMT, education, medication, psychology	44 [3925]	Meta-analysis	Random	I^2^	Pneumonia, total LOS, mortality	ICU LOS; QoL; ET/FC; POCs; safety
**Thybo Karanfil & Møller (2018) [23],** **Denmark**	Elective surgery (CABG/valve)	IMT	5 [451]	Meta-analysis	Fixed + random	I^2^	Pneumonia	PPCs
**Wang et al. (2023) [28],** **China**	Elective surgery (CABG/valve) Elective TAVI	IMT	6 [925]	Meta-analysis	Random	I^2^	Total LOS	MVT; ICU LOS
**Wang et al. (2024) [30],** **China**	Elective surgery (CABG/valve) Elective TAVI	PT, IMT, education	21 [2895]	Meta-analysis	Random	I^2^	Pneumonia, total LOS	PPCs; ICU LOS
**Yau et al. (2021) [5],** **Hong Kong**	Elective surgery (CABG/valve)	PT, IMT, education	7 [726]	Meta-analysis	Random	I^2^, X^2^	Pneumonia, total LOS, mortality	POCs; ET/FC; QoL; anxiety and depression; frailty; ICU LOS; economic costs; cardiac rehab enrolment

CABG = coronary artery bypass graft; ET/FC = exercise tolerance/functional capacity; ICU = intensive care unit; IMT = inspiratory muscle training; LOS = length of stay; MVT = mechanical ventilation time; NR = not reported; PFTs = pulmonary function tests; POCs = postoperative complications; PPCs = postoperative pulmonary complications; PT = physical therapy; QoL = quality of life; SR = systematic review. * Number of participants approximated, as study includes mixed pre-/post-IMT cohorts.

### 3.3. Methodological Quality of Included Reviews

Methodological quality, assessed using AMSTAR 2 [16], is summarised in Appendix C. Of the 17 included reviews, one was rated high-quality [31] and one moderate-quality [21], while three were rated low-quality [7,22,29] and the remainder critically low [4,5,18,20,23,24,25,26,27,28,30,32]. The high prevalence of critically low ratings was primarily driven by deficiencies in key AMSTAR 2 domains, particularly lack of protocol registration, the incomplete reporting of excluded studies and the inadequate assessment of publication bias.

Common limitations included the incomplete reporting of excluded studies [4,5,7,18,20,22,23,24,25,26,27,28,29,30,32], lack of protocol registration [4,20,24,25,27,28,30,32], limited duplication of study selection or data extraction [4,7,24,25,30], and inadequate consideration of risk of bias [4,20,23] or publication bias [4,5,18,20,23,24,25,26,27,28,30,32]. In contrast, most reviews clearly defined their research question, described the included studies adequately, and used appropriate meta-analytic methods where pooling was undertaken [4,5,7,18,20,21,22,23,24,25,26,27,28,29,30,31,32].

### 3.4. Certainty of Evidence

Given the substantial overlap among the included reviews, certainty of evidence was estimated using one representative meta-analysis per outcome rather than across all pooled analyses. Using this approach, certainty was judged moderate for postoperative pneumonia, moderate for hospital LOS, and low for mortality (Table 2).

### 3.5. Nature of Prehabilitation Interventions

Prehabilitation interventions were heterogeneous in content, duration and delivery.

#### 3.5.1. Respiratory-Focused (IMT) Interventions

IMT was the most consistently studied modality and formed the basis of several cardiac surgery-specific meta-analyses [18,20,23,24,28,31,32]. IMT-based interventions demonstrated the most consistent effects, particularly for postoperative pneumonia, with relative risk reductions of approximately 55–62% across multiple meta-analyses.

#### 3.5.2. Multimodal Prehabilitation

Multimodal interventions incorporating exercise [4,5,7,21,22,25,26,27,29,30], education [5,7,25,26,27,29,30] and psychological support [29] demonstrated more variable effects, particularly on hospital LOS, suggesting broader but less targeted impacts on recovery.

### 3.6. Evidence from Pooled Analyses

Most evidence evaluating prehabilitation before cardiac procedures derives from systematic reviews and meta-analyses of relatively small, randomised trials. These predominantly involved mixed cardiac surgical populations, most commonly CABG or valve surgery [4,5,7,18,20,21,22,23,24,25,26], with some more recent analyses also including transcatheter cardiac interventions [27,28,29,30].

#### 3.6.1. Postoperative Pneumonia

Postoperative pneumonia was the most consistently reported outcome in pooled analyses evaluating prehabilitation before cardiac surgery and cardiac interventions. Eight reviews reported pooled analyses of postoperative pneumonia [5,7,18,21,23,29,30,31], the majority of which demonstrated statistically significant effect estimates favouring prehabilitation (Table 3) [7,18,21,23,29,30,31].

Reported effect sizes were broadly consistent, with relative risks typically ranging from 0.38 to 0.45, corresponding to an estimated 55–62% relative reduction in postoperative pneumonia. Odds ratios were of similar magnitude and direction, with one outlier study reporting a larger effect [7]. Most results were statistically significant (*p* < 0.05) and were most consistent in studies evaluating IMT [18,21,30]. An informal sensitivity assessment was undertaken within the narrative synthesis by comparing effect estimates across higher-quality reviews (high and moderate AMSTAR 2 ratings). Restriction to higher-quality reviews (high and moderate AMSTAR 2 ratings) did not materially alter effect estimates for postoperative pneumonia, with relative risks remaining approximately 0.44–0.45. Similarly, analyses stratified by intervention type demonstrated that the observed reduction in pneumonia was primarily driven by IMT, with more consistent effects compared with multimodal interventions. These findings suggest that the overall conclusions are robust to variations in methodological quality and intervention design.

#### 3.6.2. Hospital Length of Stay

Hospital LOS was reported in 15 pooled analyses [4,5,7,18,20,21,22,24,25,26,27,28,29,30,32]. Results for LOS were more variable. Overall, the direction of effect favoured prehabilitation, with reductions typically ranging from approximately 0.5 to 3 days. Several analyses demonstrated statistically significant reductions, while others did not (Table 4).

#### 3.6.3. Mortality

Six pooled analyses reported mortality outcomes [5,7,18,21,22,29]. Although most point estimates numerically favoured prehabilitation, pooled analyses did not demonstrate a statistically significant mortality benefit [7,18,21,22,29]. Mortality event rates were low across reviews, with small sample sizes likely contributing to the absence of a clear signal (Table 5).

## 4. Discussion

### 4.1. Principal Findings

This umbrella review identified 17 systematic reviews evaluating prehabilitation before cardiac surgery or cardiac interventions. Across pooled analyses, the most consistent benefit was a reduction in postoperative pneumonia, while hospital LOS showed a generally favourable but more variable pattern. In contrast, no clear reduction in short-term mortality was demonstrated.

GRADE assessment indicated moderate certainty for postoperative pneumonia and hospital LOS, but only low certainty for mortality. These ratings should be interpreted in the context of substantial overlap between the included reviews and the pragmatic outcome-level approach used to avoid repeated assessment of the same evidence.

The methodological quality of the included reviews was variable, with most being rated low- or critically low-confidence using AMSTAR 2. The only high-quality review was published in 2015 and therefore predates more recent studies in this field [31]. The predominance of critically low-quality reviews is consistent with other applications of AMSTAR 2, which applies rigorous criteria to the assessment of systematic reviews [16].

Heterogeneity in patient populations, procedural types, intervention design and outcome definitions represents an important source of variation across analyses. Prehabilitation interventions were diverse, and the effects of individual components could not be disentangled.

Despite these limitations, a consistent directional signal was observed for respiratory outcomes. Postoperative pneumonia demonstrated the most reproducible effect across meta-analyses, with pooled estimates suggesting an approximately 55–62% relative reduction in risk, particularly in studies evaluating IMT. Although relative reductions appear substantial, the absolute risk reduction is more modest due to low baseline event rates. Based on pooled estimates, this corresponds to an absolute reduction of approximately 56 cases per 1000 patients and a number needed to treat (NNT) of 18, with a plausible range of 14 to 46 based on confidence intervals. This suggests that prehabilitation may provide the greatest absolute benefit in higher-risk patients, where baseline rates of postoperative pneumonia are higher. Patients with reduced baseline respiratory reserve, frailty or elevated pulmonary risk may derive the greatest benefit, although this remains insufficiently explored in current evidence.

In contrast, reductions in hospital LOS were more variable in magnitude, although the overall direction of effect generally favoured prehabilitation. This variability likely reflects the multifactorial nature of LOS, which is influenced not only by physiological recovery but also by institutional practices, discharge pathways and healthcare system factors.

IMT emerged as the most consistently evaluated intervention across the included literature and showed the most reproducible signal of benefit for postoperative pneumonia. A favourable association with hospital length of stay was also seen in several IMT-based analyses, although LOS findings were more variable overall.

No significant reduction in short-term mortality was observed, although outcome timepoints varied across studies. This likely reflects low event rates and insufficient statistical power, suggesting that mortality may not be an appropriate primary endpoint for evaluating prehabilitation in contemporary cardiac surgery populations. Importantly, the absence of a mortality signal does not diminish the clinical relevance of prehabilitation. Reductions in postoperative pneumonia and modest decreases in hospital LOS represent meaningful improvements for patients undergoing cardiac procedures.

### 4.2. Interpretation in the Wider Context of the Literature

These findings should be interpreted in the context of the broader literature, characterised by heterogeneity in intervention design, inconsistent definitions of prehabilitation, and increasing but variable implementation in clinical practice [9,10]. While previous work has highlighted these challenges, this umbrella review provides a structured synthesis across overlapping systematic reviews, incorporating assessment of methodological quality and review overlap. In doing so, it clarifies that the most consistent evidence of benefit relates to postoperative pneumonia, whereas effects on hospital LOS and mortality remain more uncertain.

This pattern is consistent with the wider perioperative literature, in which prehabilitation is associated with improvements in functional capacity, respiratory performance and postoperative recovery, but less consistently with hard endpoints such as mortality. Prehabilitation has been more extensively studied in cancer and orthopaedic populations, where similar findings have been observed, supporting the generalisability of these observations across surgical disciplines [81,82]. Emerging models of care, including tele-prehabilitation and digitally supported home-based programmes, may address barriers to implementation such as travel limitations and resource constraints while improving adherence and scalability [83]. Although cost-effectiveness was inconsistently reported, reductions in pneumonia and LOS suggest potential economic benefits, particularly in high-risk populations.

### 4.3. Limitations

Several limitations should be considered when interpreting these findings. The available evidence is largely derived from relatively small trials, and there was substantial overlap of primary studies across the included reviews, meaning that consistency across meta-analyses does not necessarily represent independent replication.

Prehabilitation interventions were heterogeneous in design and delivery, limiting direct comparability across studies. In addition, most systematic reviews were rated low- or critically low-quality. Despite inclusion in the review scope, evidence for structural heart interventions remains limited. This represents a critical research gap, particularly given the older and frailer populations undergoing percutaneous procedures such as transcatheter aortic valve implantation (TAVI).

### 4.4. Future Directions

Future research should prioritise adequately powered randomised controlled trials with standardised outcome definitions. More rigorous systematic reviews are also required, with improved methodological quality and reporting. Future work should extend beyond CABG and valve populations to include structural heart interventions and adults with congenital heart disease, where evidence remains relatively limited and less well characterised. Future trials should prioritise outcomes that are more responsive to prehabilitation, including functional recovery outcomes (e.g., 6 min walk distance), respiratory complications, quality of recovery, independence and patient-reported outcome measures (PROMs), which may better capture the benefits of prehabilitation than mortality endpoints.

## 5. Conclusions

Prehabilitation is associated across pooled meta-analyses with a reduction in postoperative pneumonia and, less consistently, shorter hospital LOS in patients undergoing cardiac surgery and structural interventions. The most consistent signal of benefit is observed with IMT. However, the evidence base remains limited by heterogeneity, overlap between studies, and predominantly low methodological quality. Further high-quality, adequately powered trials with standardised intervention protocols and outcome definitions are required to define the role of prehabilitation in contemporary practice.

## Figures and Tables

**Table 2:** GRADE table.

Outcome	Relative Effect (95% CI)	Anticipated Absolute Effects	No. of Participants [Studies]	Certainty of Evidence (GRADE)	Comments
**Pneumonia**	RR 0.44 (0.25 to 0.78)	Risk with control: 100 per 1000 Risk with intervention: 44 per 1000 (25–78) NNT 18 (range 14–46)	645 [6]	**Moderate**	Cardiac-only evidence (Cursino de Moura 2024) [18]
**Hospital LOS**	MD −1.77 days (−2.65 to −0.89)	Mean LOS reduced by 1.77 days	925 [6]	**Moderate**	Consistent reduction (Wang 2023) [28]
**Mortality**	OR 1.30 (0.28 to 5.95)	No clear difference	532 [4]	Low	Imprecise, rare events (Yau 2021) [5]

CI = confidence interval; LOS = length of stay; MD = mean difference; NNT = number needed to treat; OR = odds ratio; RR = relative risk. NNT was calculated from the absolute risk reduction, derived by applying the pooled relative risk to the baseline event rate. The reported range reflects uncertainty in the relative effect (95% confidence interval).

**Table 3:** Pneumonia.

Study	Population	Model Used	Type of Intervention	Dates of Included Studies	Number of Studies (Participants) [Reference]	Pneumonia Events (Intervention/ Control)	Heterogeneity	Effect Size [95% CI]
**Cursino de Moura et al. (2024) [18]**	Elective surgery (CABG/valve)	Meta-analysis	IMT	1998–2019	6 (645) [33,34,35,36,37,38]	16/36	I^2^ = 0%	**RR 0.44 [0.25–0.78]**
**Hulzebos et al. (2012) [21]**	Elective surgery (CABG/valve)	Cochrane review	IMT	1998–2011	5 (448) [33,34,36,37,38]	13/29	I^2^ = 0%	**RR 0.45 [0.24–0.83]** **(*p* = 0.01)**
**Katsura et al. (2015) [31]**	Elective surgery (CABG/valve)	Cochrane review	Education, IMT	1998–2011	5 (448) [33,34,36,37,38]	13/29	I^2^ = 0%	**RR 0.44 [0.23–0.83]** **(*p* = 0.01)**
**Steinmetz et al. (2023) [7]**	Elective surgery (CABG/valve)	Meta-analysis	Education, PT, IMT	2008–2016	2 (82) [39,40]	NR	I^2^ = 0%	RR 0.12 [0.02–1.00] (*p* = 0.05)
**Steinmetz et al. (2026) [29]**	Elective surgery (CABG/valve) Elective TAVI	Meta-analysis	Education, PT, IMT	1998–2022	5 (729) [33,38,39,41,42]	18/53	I^2^ = 0%	**OR 0.33 [0.15–0.72]** **(*p* = 0.017)**
**Thybo Karanfil & Møller (2018) [23]**	Elective surgery (CABG/valve)	Meta-analysis	Education, IMT	1998–2011	5 (448) [33,34,36,37,38]	13/29	I^2^ = 0%	**RR 0.44 [0.23–0.83]** **(*p* = 0.01)**
**Wang et al. (2024) [30]**	Elective surgery (CABG)	Meta-analysis	IMT	1998–2019	7 (880) [33,34,35,36,37,38,42]	24/54	I^2^ = 0%	**OR 0.41 [0.25–0.67]** **(*p* = 0.0004)**
**Yau et al. (2021) [5]**	Elective surgery (CABG/valve)	Meta-analysis	Education, PT, IMT	2008–2016	2 (82) [39,40]	NR	I^2^ = 67%	RR 0.38 [0.01–14.64]

CABG = coronary artery bypass graft; CI = confidence interval; IMT = inspiratory muscle training; OR = odds ratio; PT = physical therapy; RR = risk ratio; TAVI = transcatheter aortic valve implantation.

**Table 4:** Total hospital length of stay.

Study	Population	Model Used	Type of Intervention	Dates of Included Studies	Number of Studies (Participants) [Reference]	Heterogeneity	Effect Size MD (Days) [95% CI]
**Cursino de Moura et al. (2024) [18]**	Elective surgery (CABG/valve)	Meta-analysis	IMT	2006–2019	4 (531) [33,34,35,37]	I^2^ = 0%	**−1.7 [−2.4 to −1.1]**
**Elbadrawy et al. (2025) [27]**	Elective surgery (CABG/valve)	Meta-analysis	Education, PT, IMT	2000–2022	5 (695) [37,43,44,45,46]	I^2^ = 87%	**−2.92 [−4.52 to −1.31]** **(*p* = 0.0004)**
**Gomes Neto et al. (2017) [20]**	Elective surgery (CABG)	Meta-analysis	IMT	2006	2 (302) [33,37]	I^2^ = 0%	**−2.04 [−3.37 to −0.72]** **(*p* = 0.003)**
**Hulzebos et al. (2012) [21]**	Elective surgery (CABG/valve)	Cochrane review	IMT	1998–2006	3 (347) [33,37,47]	I^2^ = 64%	**−3.21 [−5.73 to −0.69]** **(*p* = 0.01)**
**Hurtado-Borrego et al. (2025) [22]**	Elective surgery (CABG/valve)	Meta-analysis	Education, PT, IMT	2008–2025	5 (501) [39,48,49,50,51]	I^2^ = 84%	−0.63 [−1.44 to 0.18]
**Kendall et al. (2018) [32]**	Elective surgery (CABG)	Meta-analysis	IMT	NR	5	I^2^ = 0%	**−1.19 [−1.88 to −0.49]**
**Marmelo et al. (2018) [4]**	Elective surgery (CABG/valve)	Meta-analysis	Education, PT, IMT	2000–2015	8 (945) [33,37,42,43,50,52,53,54]	I^2^ = 93%	−0.56 [−1.13 to 0.01] (*p* = 0.05)
**Rodrigues et al. (2021) [24]**	Elective surgery (CABG/valve)	Meta-analysis	IMT	2005–2019	7 (1050) [33,35,37,53,55,56,57]	I^2^ = 34%	−0.81 [−1.38 to −0.48]
**Shahood et al. (2022) [26]**	Elective surgery (CABG/valve)	Meta-analysis	PT, IMT	2006–2019	8 (1228) [33,35,37,42,52,53,54,56]	I^2^ = 41%	**−1.02 [−1.42 to −0.61]** **(*p* < 0.00001)**
**Snowdon et al. (2014) [25]**	Elective surgery (CABG)	Meta-analysis	Education, PT, IMT	1992–2009	10 (1573) [33,37,47,58,59,60,61,62,63,64]	I^2^ = 76%	−0.55 [−1.32 to 0.23]
**Steinmetz et al. (2023) [7]**	Elective surgery (CABG/valve)	Meta-analysis	Education, PT, IMT	2000–2020	6 (621) [39,40,43,49,50,65]	I^2^ = 92%	**−1.00 [−1.78 to −0.23]** **(*p* = 0.01)**
**Steinmetz et al. (2026) [29]**	Elective surgery (CABG/valve) Elective TAVI	Meta-analysis	Medication, psychological, education, PT, IMT	1992–2024	18 (1531) [33,39,42,43,48,49,50,57,58,66,67,68,69,70,71,72,73,74]	I^2^ = 94%	**−0.95 [−1.77 to −0.13]** **(*p* = 0.026)**
**Wang et al. (2023) [28]**	Elective surgery (CABG/valve) Elective TAVI	Meta-analysis	Education, IMT	2006–2021	6 (925) [33,35,42,52,53,75]	I^2^ = 17%	**−1.77 [−2.41 to −1.12]** **(*p* < 0.00001)**
**Wang et al. (2024) [30]**	Elective surgery (CABG)	Meta-analysis	IMT	1998–2019	8 (924) [33,34,35,37,42,47,52,53]	I^2^ = 52%	**−1.57 [−2.33 to −0.81]** **(*p* < 0.0001)**
	Elective surgery (CABG) Elective TAVI	Meta-analysis	PT	1994–2021	5 (607) [39,43,49,75,76]	I^2^ = 94%	**−1.82 [−3.38 to −0.27]** **(*p* = 0.02)**
**Yau et al. (2021) [5]**	Elective surgery (CABG/valve)	Meta-analysis	Education, PT	2000–2014	2 (235) [43,50]	I^2^ = 0%	**−0.62 [−0.93 to −0.32]**

CABG = coronary artery bypass graft; CI = confidence interval; IMT = inspiratory muscle training; LOS = length of stay; MD = mean difference; NR = not reported; PT = physical therapy; TAVI = transcatheter aortic valve implantation. Mean differences were aligned so that negative values indicate a reduction in the outcome (e.g., shorter LOS).

**Table 5:** Mortality.

Study	Population	Model Used	Type of Intervention	Dates of Included Studies	Number of Studies (Participants) [Reference]	Mortality Events (Intervention/ Control)	Heterogeneity	Effect Size [95% CI]
**Cursino de Moura et al. (2024) [18]**	Elective surgery (CABG/valve)	Meta-analysis	IMT	2006–2009	2 (306) [33,36]	3 / 5	I^2^ = 71%	RR 0.63 [0.17–2.33]
**Hulzebos et al. (2012) [21]**	Elective surgery (CABG/valve)	Cochrane review	PT, IMT	2000–2009	3 (552) [33,36,43]	3 / 5	I^2^ = 71%	RR 0.66 [0.02–18.48] (*p* = 0.81)
**Hurtado-Borrego et al. (2025) [22]**	Elective surgery (CABG/valve)	Meta-analysis	PT, IMT	2011–2025	5 (523) [48,49,51,77,78]	NR	I^2^ = 0%	OR 0.67 [0.25–1.77] (*p* = 0.42)
**Steinmetz et al. (2023) [7]**	Elective surgery (CABG/valve)	Meta-analysis	Education, PT, IMT	2000–2020	4 (531) [39,40,43,65]	NR	I^2^ = 59%	OR 0.54 [0.14–2.06] (*p* = 0.36)
**Steinmetz et al. (2026) [29]**	Elective surgery (CABG/valve) Elective TAVI	Meta-analysis	Medication, psychological, PT, IMT	2005–2023	5 (770) [33,48,70,79,80]	33/45	I^2^ = 23%	OR 0.82 [0.27–2.46] (*p* = 0.64)
**Yau et al. (2021) [5]**	Elective surgery (CABG/valve)	Meta-analysis	Education, PT, IMT	2000–2016	4 (532) [39,40,43,64]	4/3	I^2^ = 0%	OR 1.30 [0.28–5.95]

CABG = coronary artery bypass graft; CI = confidence interval; IMT = inspiratory muscle training; OR = odds ratio; PT = physical therapy; RR = risk ratio; TAVI = transcatheter aortic valve implantation.

## Data Availability

No new primary data were generated in this study. All data analysed were extracted from published studies cited in the article.

## Abbreviations

The following abbreviations are used in this manuscript:

AMSTAR 2	A Measurement Tool to Assess Systematic Reviews 2
CABG	Coronary artery bypass graft
CCA	Corrected covered area
CI	Confidence interval
ET	Exercise tolerance
FC	Functional capacity
GRADE	Grading of Recommendations Assessment, Development and Evaluation
ICU	Intensive care unit
IMT	Inspiratory muscle training
LOS	Length of stay
MD	Mean difference
MVT	Mechanical ventilation time
NNT	Number needed to treat
NR	Not reported
OR	Odds ratio
OSF	Open Science Framework
PFTs	Pulmonary function tests
PICO	Population, Intervention, Comparator, Outcome
POCs	Postoperative complications
PPCs	Postoperative pulmonary complications
PRISMA	Preferred Reporting Items for Systematic Reviews and Meta-Analyses
PROMs	Patient-reported outcome measures
PT	Physical therapy
QoL	Quality of life
RoB	Risk of bias
RR	Risk ratio
SR	Systematic review
TAVI	Transcatheter aortic valve implantation

## Appendix A. PRISMA 2020 Checklist

**Section and Topic**	**Item #**	**Checklist Item**	**Location Where Item Is Reported**
**TITLE**			
Title	1	Identify the report as a systematic review.	Page 1, Lines 1–3
**ABSTRACT**			
Abstract	2	See the PRISMA 2020 for Abstracts checklist.	Pages 30 & 31, Appendix D
**INTRODUCTION**			
Rationale	3	Describe the rationale for the review in the context of existing knowledge.	Page 2, Lines 41–67
Objectives	4	Provide an explicit statement of the objective(s) or question(s) the review addresses.	Page 2, Lines 68–77
**METHODS**			
Eligibility criteria	5	Specify the inclusion and exclusion criteria for the review and how studies were grouped for the syntheses.	Page 3, Lines 90–99
Information sources	6	Specify all databases, registers, websites, organisations, reference lists and other sources searched or consulted to identify studies. Specify the date when each source was last searched or consulted.	Page 3, Lines 101–107
Search strategy	7	Present the full search strategies for all databases, registers and websites, including any filters and limits used.	Page 28, Appendix B
Selection process	8	Specify the methods used to decide whether a study met the inclusion criteria of the review, including how many reviewers screened each record and each report retrieved, whether they worked independently, and if applicable, details of automation tools used in the process.	Page 3, Lines 108–120
Data collection process	9	Specify the methods used to collect data from reports, including how many reviewers collected data from each report, whether they worked independently, any processes for obtaining or confirming data from study investigators, and if applicable, details of automation tools used in the process.	Page 3, Lines 108–120
Data items	10a	List and define all outcomes for which data were sought. Specify whether all results that were compatible with each outcome domain in each study were sought (e.g., for all measures, time points, analyses), and if not, the methods used to decide which results to collect.	Page 3, Lines 90–99
	10b	List and define all other variables for which data were sought (e.g., participant and intervention characteristics, funding sources). Describe any assumptions made about any missing or unclear information.	Pages 7 & 8, Table 1
Study risk of bias assessment	11	Specify the methods used to assess risk of bias in the included studies, including details of the tool(s) used, how many reviewers assessed each study and whether they worked independently, and if applicable, details of automation tools used in the process.	Page 3, Lines 108–130
Effect measures	12	Specify for each outcome the effect measure(s) (e.g., risk ratio, mean difference) used in the synthesis or presentation of results.	Page 4, Lines 156–165
Synthesis methods	13a	Describe the processes used to decide which studies were eligible for each synthesis (e.g., tabulating the study intervention characteristics and comparing against the planned groups for each synthesis (item #5)).	Pages 7 & 8, Table 1
	13b	Describe any methods required to prepare the data for presentation or synthesis, such as handling of missing summary statistics, or data conversions.	NA
	13c	Describe any methods used to tabulate or visually display results of individual studies and syntheses.	NA
	13d	Describe any methods used to synthesize results and provide a rationale for the choice(s). If meta-analysis was performed, describe the model(s), method(s) to identify the presence and extent of statistical heterogeneity, and software package(s) used.	NA
	13e	Describe any methods used to explore possible causes of heterogeneity among study results (e.g., subgroup analysis, meta-regression).	Page 4, Lines 153–155
	13f	Describe any sensitivity analyses conducted to assess robustness of the synthesized results.	NA
Reporting bias assessment	14	Describe any methods used to assess risk of bias due to missing results in a synthesis (arising from reporting biases).	Pages 3 & 4, Lines 121–152
Certainty assessment	15	Describe any methods used to assess certainty (or confidence) in the body of evidence for an outcome.	Pages 3 & 4, Lines 131–152
**RESULTS**			
Study selection	16a	Describe the results of the search and selection process, from the number of records identified in the search to the number of studies included in the review, ideally using a flow diagram.	Page 4, Lines 166–172
	16b	Cite studies that might appear to meet the inclusion criteria, but which were excluded, and explain why they were excluded.	NA
Study characteristics	17	Cite each included study and present its characteristics.	Pages 7 & 8, Table 1
Risk of bias in studies	18	Present assessments of risk of bias for each included study.	Pages 29 & 30, Appendix C
Results of individual studies	19	For all outcomes, present, for each study: (a) summary statistics for each group (where appropriate) and (b) an effect estimate and its precision (e.g., confidence/credible interval), ideally using structured tables or plots.	Pages 12-15, Table 3, Table 4 and Table 5
Results of syntheses	20a	For each synthesis, briefly summarise the characteristics and risk of bias among contributing studies.	Pages 10 & 11, Lines 230–279
	20b	Present results of all statistical syntheses conducted. If meta-analysis was done, present for each the summary estimate and its precision (e.g., confidence/credible interval) and measures of statistical heterogeneity. If comparing groups, describe the direction of the effect.	NA
	20c	Present results of all investigations of possible causes of heterogeneity among study results.	Pages 5 & 6, Lines 198–211
	20d	Present results of all sensitivity analyses conducted to assess the robustness of the synthesized results.	Page 10, Lines 218–222
Reporting biases	21	Present assessments of risk of bias due to missing results (arising from reporting biases) for each synthesis assessed.	NA
Certainty of evidence	22	Present assessments of certainty (or confidence) in the body of evidence for each outcome assessed.	Page 10, Lines 218–222
**DISCUSSION**			
Discussion	23a	Provide a general interpretation of the results in the context of other evidence.	Page 17, Lines 345–364
	23b	Discuss any limitations of the evidence included in the review.	Page 17, Lines 365–375
	23c	Discuss any limitations of the review processes used.	Page 17, Lines 365–375
	23d	Discuss implications of the results for practice, policy, and future research.	Page 17, Lines 376–386
**OTHER INFORMATION**			
Registration and protocol	24a	Provide registration information for the review, including register name and registration number, or state that the review was not registered.	Page 2, Lines 82–89
	24b	Indicate where the review protocol can be accessed, or state that a protocol was not prepared.	Page 2, Line 86
	24c	Describe and explain any amendments to information provided at registration or in the protocol.	NA
Support	25	Describe sources of financial or non-financial support for the review, and the role of the funders or sponsors in the review.	Page 18, Line 400
Competing interests	26	Declare any competing interests of review authors.	Page 18, Line 410
Availability of data, code and other materials	27	Report which of the following are publicly available and where they can be found: template data collection forms; data extracted from included studies; data used for all analyses; analytic code; any other materials used in the review.	NA

## Appendix B

Search strategies:

PubMED:

(prehabilitation[Title/Abstract] OR preoperative rehabilitation[Title/Abstract] OR pre-operative rehabilitation[Title/Abstract] OR preoperative exercise[Title/Abstract] OR exercise training[Title/Abstract] OR inspiratory muscle training[Title/Abstract] OR respiratory muscle training[Title/Abstract] OR IMT[Title/Abstract] OR physiotherapy[Title/Abstract] OR chest physiotherapy[Title/Abstract] OR respiratory physiotherapy[Title/Abstract] OR breathing exercises[Title/Abstract]) AND (Cardiac Surgical Procedures[Mesh] OR Heart Valve Surgical Procedures[Mesh] OR Coronary Artery Bypass[Mesh] OR cardiac surgery[Title/Abstract] OR CABG[Title/Abstract] OR valve surgery[Title/Abstract] OR heart valve surgery[Title/Abstract] OR TAVI[Title/Abstract] OR TAVR[Title/Abstract]) AND (“systematic review”[Title/Abstract] OR “meta-analysis”[Title/Abstract] OR “meta analysis”[Title/Abstract] OR “pooled analysis”[Title/Abstract] OR systematic review[Publication Type] OR meta-analysis[Publication Type])

EMBASE:

(prehabilitation OR preoperative rehabilitation OR pre-operative rehabilitation OR preoperative exercise OR exercise training OR inspiratory muscle training OR respiratory muscle training OR IMT OR physiotherapy OR chest physiotherapy OR respiratory physiotherapy OR breathing exercises).ti,ab,kw. AND (exp cardiac surgery/ OR exp heart valve surgery/ OR exp coronary artery bypass/ OR (cardiac surgery OR CABG OR valve surgery OR heart valve surgery OR TAVI OR TAVR).ti,ab,kw.) AND (systematic review OR meta-analysis OR meta analysis OR pooled analysis).ti.

Cochrane Central Library:

(‘prehabilitation’ OR ‘preoperative rehabilitation’ OR ‘pre-operative rehabilitation’ OR ‘preoperative exercise’ OR ‘exercise training’ OR ‘inspiratory muscle training’ OR ‘respiratory muscle training’ OR IMT OR ‘rehabilitation’ OR ‘exercise therapy’) AND (‘cardiac surgery’ OR ‘heart surgery’ OR ‘coronary artery bypass’/exp OR CABG OR ‘valve surgery’ OR ‘transcatheter aortic valve implantation’ OR TAVI OR TAVR OR ‘structural heart’)—Title or abstract filtering on the above.

Filter: Cochrane Reviews

## Appendix C. AMSTAR 2 Methodological Appraisal Table

**Study**	**PICO**	**Protocol ***	**Design Choice**	**Search ***	**Selection Duplicate**	**Extraction Duplicate**	**Excluded Studies ***	**Included Study** **Details**	**RoB Tool ***	**Funding of Primary Studies**	**Meta Methods ***	**RoB Impact on Synthesis**	**RoB in Interpretation ***	**Heterogeneity**	**Publication Bias ***	**Review COI**	**Overall**
Cursino de Moura 2024 [18]	Yes	Yes	Yes	Yes	Yes	Yes	No	Yes	Yes	No	Yes	No	Partial	Yes	No	Yes	Critically low
Elbadrawy 2025 [27]	Yes	No	Yes	Partial	Partial	Yes	No	Yes	Yes	Partial	Yes	No	Partial	Yes	No	Yes	Critically low
Gomes Neto 2017 [20]	Yes	No	Yes	Partial	Yes	Yes	No	Yes	No	No	Yes	No	No	Yes	No	Yes	Critically low
Hulzebos 2012 [21]	Yes	Yes	Yes	Yes	Yes	Yes	Yes	Yes	Yes	No	Yes	No	Yes	Yes	Partial	Yes	Moderate
Hurtado-Borrego 2025 [22]	Yes	Yes	Yes	Yes	Yes	Yes	No	Yes	Yes	No	Yes	No	Yes	Yes	Yes	Yes	Low
Katsura 2015 [31]	Yes	Yes	Yes	Yes	Yes	Yes	Yes	Yes	Yes	No	Yes	Yes	Yes	Yes	Yes	Yes	High
Kendall 2018 [32]	Yes	No	Yes	Partial	Yes	No	No	Yes	No	No	Yes	No	Partial	Yes	Yes	Yes	Critically low
Marmelo 2018 [4]	Yes	No	Yes	Partial	No	No	No	Yes	Yes	No	Yes	No	No	Yes	No	Yes	Critically low
Rodrigues 2021 [24]	Yes	No	Yes	Partial	Yes	No	No	Yes	Yes	No	Yes	No	Partial	Yes	No	Partial	Critically low
Shahood 2022 [26]	Yes	Yes	Yes	Partial	Partial	Partial	No	Yes	Yes	No	Yes	No	Partial	Yes	No	Yes	Critically low
Snowdon 2014 [25]	Yes	No	Yes	Yes	Yes	Yes	No	Yes	Yes	No	Yes	No	Partial	Yes	No	Yes	Critically low
Steinmetz 2023 [7]	Yes	Yes	Yes	Partial	Yes	No	No	Yes	Yes	No	Yes	No	Yes	Yes	Partial	Yes	Low
Steinmetz 2026 [29]	Yes	Yes	Yes	Yes	Yes	Yes	No	Yes	Yes	No	Yes	No	Yes	Yes	Partial	Yes	Low
Thybo Karanfil 2018 [23]	Yes	Yes	Yes	Yes	Yes	Yes	No	Yes	Yes	No	Yes	No	No	Yes	No	Yes	Critically low
Wang 2023 [28]	Yes	No	Yes	Partial	Yes	Yes	No	Yes	Yes	No	Yes	No	Partial	Yes	No	Partial	Critically low
Wang 2024 [30]	Yes	No	Yes	Partial	Yes	No	No	Yes	Yes	No	Yes	No	Partial	Yes	No	Yes	Critically low
Yau 2021 [5]	Yes	Yes	Yes	Yes	Yes	Yes	No	Yes	Yes	No	Yes	No	Yes	Yes	No	Yes	Critically low
* Critical AMSTAR 2 domains.																	

## Appendix D. PRISMA 2020 Abstract Checklist

**Topic**	**No.**	**Item**	**Reported?**
**TITLE**			
**Title**	1	Identify the report as a systematic review.	Yes
**BACKGROUND**			
**Objectives**	2	Provide an explicit statement of the main objective(s) or question(s) the review addresses.	No
**METHODS**			
**Eligibility criteria**	3	Specify the inclusion and exclusion criteria for the review.	No
**Information sources**	4	Specify the information sources (e.g., databases, registers) used to identify studies and the date when each was last searched.	No
**Risk of bias**	5	Specify the methods used to assess risk of bias in the included studies.	Yes
**Synthesis of results**	6	Specify the methods used to present and synthesize results.	No
**RESULTS**			
**Included studies**	7	Give the total number of included studies and participants and summarise relevant characteristics of studies.	Yes
**Synthesis of results**	8	Present results for main outcomes, preferably indicating the number of included studies and participants for each. If meta-analysis was done, report the summary estimate and confidence/credible interval. If comparing groups, indicate the direction of the effect (i.e., which group is favoured).	Yes
**DISCUSSION**			
**Limitations of evidence**	9	Provide a brief summary of the limitations of the evidence included in the review (e.g., study risk of bias, inconsistency and imprecision).	Yes
**Interpretation**	10	Provide a general interpretation of the results and important implications.	Yes
**OTHER**			
**Funding**	11	Specify the primary source of funding for the review.	No
**Registration**	12	Provide the register name and registration number.	No

## Appendix E

**Overlap Matrix—Pneumonia (■ = Primary Study Included in Review)**											
**Systematic Review**	**Weiner 1998 [38]**		**Hulzebos 2006 (Helders) [33]**	**Hulzebos 2006 (van Meeteren) [37]**	**Ferreira 2009 [36]**	**Carvalho 2011 [34]**	**Herdy 2008 [39]**	**Chen 2019 [35]**	**Stammers 2016 [40]**	**Valkenet 2017 [42]**	**Argunova 2022 [41]**
Cursino de Moura 2024 [18]	■		■	■	■	■		■			
Hulzebos 2012 [21]	■		■	■	■	■					
Katsura 2015 [31]	■		■	■	■	■					
Steinmetz 2023 [7]							■		■		
Steinmetz 2026 [29]	■		■				■			■	■
Thybo Karanfil & Møller 2018 [23]	■		■	■	■	■					
Wang 2024 [30]	■		■	■	■	■		■		■	
Yau 2021 [5]							■		■		
**Frequency**	**6**		**6**	**5**	**5**	**5**	**3**	**2**	**2**	**2**	**1**
Note: Frequencies represent the number of systematic reviews in which each primary study appears. Columns are ordered by frequency (highest to lowest). This matrix illustrates which primary studies were included in each systematic review reporting pneumonia (■ = included). The clustering of squares around specific studies demonstrates substantial overlap, with multiple reviews drawing on the same core set of evidence.											

**Overlap Matrix—Length of Stay (■ = Primary Study Included in Review)**											
**Systematic Reviews**	**Hulzebos 2006** **(Helders)** [33]	**Hulzebos** **(van Meeteren)** [37]	**Arthur 2000** [43]	**Chen 2019** [35]	**Sawatzky 2014** [50]	**Sobrinho 2014** [53]	**Valkenet 2017** [42]	**Herdy 2008** [39]	**Rosenfeldt 2011** [49]	**Savci 2011** [52]	**Rajendran 1998** [47]
Cursino de Moura 2024 [18]	■	■		■							
Elbadrawy 2025 [27]		■	■								
Gomes Neto 2017 [20]	■	■									
Hulzebos 2012 [21]	■	■									■
Hurtado-Borrego 2025 [22]					■			■	■		
Kendall 2018 [32]											
Marmelo 2018 [4]	■	■	■		■	■	■			■	
Rodrigues 2021 [24]	■	■		■		■					
Shahood 2022 [26]	■	■		■		■	■			■	
Snowdon 2014 [25]	■	■									■
Steinmetz 2023 [7]			■		■			■	■		
Steinmetz 2026 [29]	■		■		■		■	■	■		
Wang 2023 [28]	■			■		■	■			■	
Wang 2024 [30]	■	■	■	■		■	■	■	■	■	■
Yau 2021 [5]			■		■						
**Frequency**	**10**	**9**	**6**	**5**	**5**	**5**	**5**	**4**	**4**	**4**	**3**
Note: Frequencies represent the number of systematic reviews in which each primary study appears. Columns are ordered by frequency (highest to lowest). Due to volume of papers only those included more than twice are included. This matrix illustrates which primary studies were included in each systematic review reporting LOS (■ = included). The clustering of squares around specific studies demonstrates substantial overlap, with multiple reviews drawing on the same core set of evidence.											

**Overlap Matrix—Mortality (■ = Primary Study Included in Review)**															
**Systematic Reviews**	**Arthur 2000** [43]	**Hulzebos 2006** [33]	**Akowuah 2023** [48]	**Ferreira 2009** [36]	**Herdy 2008** [39]	**Stammers 2016** [40]	**Furze 2009** [64]	**Lopez-Hernandez 2023** [78]	**Rideout 2012** [79]	**Rief 2017** [80]	**Rosenfeldt 2005** [70]	**Rosenfeldt 2011** [49]	**Steinmetz 2020** [65]	**Waite 2017** [77]	**Yau 2025** [51]
Cursino de Moura 2024 [18]		■		■											
Hulzebos 2012 [21]	■	■		■											
Hurtado-Borrego 2025 [22]			■					■				■		■	■
Steinmetz 2023 [7]	■				■	■							■		
Steinmetz 2026 [29]		■	■						■	■	■				
Yau 2021 [5]	■				■	■	■								
**Frequency**	**3**	**3**	**2**	**2**	**2**	**2**	**1**	**1**	**1**	**1**	**1**	**1**	**1**	**1**	**1**
Note: Frequencies represent the number of systematic reviews in which each primary study appears. Columns are ordered by frequency (highest to lowest).															
